# Shaping ability of 2Shape and NeoNiTi rotary instruments in preparation of curved canals using micro-computed tomography

**DOI:** 10.1186/s12903-021-01961-x

**Published:** 2021-11-19

**Authors:** Ibrahim Faisal, Rajab Saif, Mona Alsulaiman, Zuhair S. Natto

**Affiliations:** 1 Department of Endodontics, Faculty of Dentistry, King Abdulaziz University, Jeddah, Saudi Arabia; 2 Department of Dental Public Health, Faculty of Dentistry, King Abdulaziz University, Jeddah, Saudi Arabia

## Abstract

**Background:**

Various systems of nickel-titanium (NiTi) instrument have long been commercially available. However, the preparation of narrow and curved root canals has always been challenging. The purpose of this study was to compare the shaping ability of two NiTi systems (2Shape and NeoNiTi) in severely curved root canals with different morphological patterns using micro-computed tomography (Micro-CT).

**Methods:**

A total of 22 human extracted permanent teeth of mandibular first molars, with the exact mesial angle of curvature of 25 and 35 degrees, according to Schneider’s technique, were distributed randomly into two groups (group I: 2Shape, group II: NeoNiTi) based on the rotary system used (n = 22). The groups were subdivided into two subgroups corresponding to the angle of canal curvature (25° and 35°) (n = 11). Canals were scanned using Micro-CT pre- and post-preparation to assess the volume of dentin removed; canal transportation; and canal centering ratio at 3, 6, and 9 mm from the apex. The Mann–Whitney U test was utilized to determine any significant differences between the two systems. The level of statistical significance was set at *p* < 0.05.

**Results:**

There was no significant difference between the two groups in volume of dentin removed; canal transportation; and centering ability for 25° and 35° canal curvatures at 3, 6, and 9 mm from the apex (coronal, middle, and apical) thirds (*p* > 0.05). At the middle third, the NeoNiTi group demonstrated a statistically significant increase in volume of dentin removed for 35° canal curvatures compared to the 2Shape group.

**Conclusion:**

Within the limitation of our in vitro study, 2Shape and NeoNiTi systems with severely curved canals were confirmed to be relatively safe in preparation and to respect original canal anatomy. Nevertheless, NeoNiTi instruments produced more centered preparation and minimal canal deviation compared to the 2Shape system.

## Background

Root canal shaping is a necessary step for successful endodontic therapy. Endodontic treatment is aimed at the elimination of periapical bacteria, which is caused by root canal disease. This is achieved by the chemo-mechanical preparation of the root canal system [1, 2]. It is essential to maintain the canal's original form as far as possible while the root canal is gradually widened from the apical to the coronal region [3].

Various systems of nickel-titanium (NiTi) instruments have been available in the market. These instruments can help in the mechanical and biological preparations [4]. Nickel-titanium (NiTi) rotary instruments produce the canal after a more centered preparation, with less deviation than other instruments [5]. However, the preparation of narrow and curved root canals has always been a challenge, due to the tendency of the canal to deviate from its normal axis after preparation [6]. Besides, the curvature introduces a complexity that influences instruments' ability to prepare and clean all root canal walls regardless of the instrumentation system used [7, 8]. These difficulties in preparation may predispose to persistent root canal infection, with a consequent risk for treatment outcome [9].

2Shape (Micro-Mega, Besancon, France) is a sequence with two shaping files in continuous rotation which have been heat treated using T Wire technology [10, 11]. According to the manufacturer, the flexibility of instruments increases user comfort [10, 11] and facilitates an excellent negotiation of curvatures with the instruments, which return to their initial shape after each use [10, 11]. The file has an asymmetrical cross-section that offers superior cleaning of the root canal walls with two main and one secondary cutting edges [10, 11]. These edges increase the cutting efficiency of the file and improve debris removal. The 2S system is composed of TS1 (25/0.04), TS2 (25/0.06), F35 (35/0.06), and F40 (40/0.04) files [10, 11].

NeoNiTi (Neolix, France) is manufactured with a wire-cut electrical discharge machining (EDM) process and is used to prepare the root canal until the apex. The advantages of this (EDM) technique of the NeoNiTi file system are minimal residual stresses, a high level of accuracy, and more advanced surface finishing [12].

The features of alloys are affected by receiving EDM processes for instance fatigue resistance [13] and surface hardness [14]. Moreover, the flexibility of these files improved compared to files in a proper heat treatment process. It has a non-homothetic rectangular cross-section and consists of one file C1 for enlargement of the coronal part and three A1 files with different tip sizes of #20, #25, and # 40 for canal preparation until the apex. It provides the ideal shape of canals to receive the gutta-percha filling [15, 16].

To our knowledge, few studies have compared instruments composed of a wire-cut electrical discharge machining (WEDM) process NiTi alloy and those composed of the T-Wire heat-treated NiTi alloy in different canal morphology. Hence, our study aimed to investigate the shaping ability of 2Shape and NeoNiTi rotary NiTi systems in the preparation of curved canals using micro-computed tomography (Micro-CT). The null hypothesis is there is no difference in shaping ability between the two experimented file systems in shaping canals with different morphological patterns.

## Methods

### Sample selection

This study was conducted with approval from the Research Ethics Committee (#113-11-20) at King Abdulaziz University, Jeddah, KSA. This is a pilot study for which a total of 22 human extracted permanent teeth of mandibular first molars with mesial curved root canals were collected from the Oral and Maxillofacial Surgery department in the faculty of dentistry, King Abdulaziz University. The teeth were extracted for periodontal or prosthodontic reasons. The patients were of unknown gender and age.

Inclusion criteria were as follows: all teeth had two separate canals in the mesial root (mesiobuccal and mesiolingual) as confirmed by digital periapical radiographs from the Carestream (CS) R4 Clinical and Practice Management Software (CS Health, Inc. Rochester, NY, USA) in a mesiodistal and buccolingual direction with two separate apical foramen (Vertucci type IV configuration). Teeth with fully formed apices and a standardized root canal curvature of 25° and 35°, measured according to Schneider’s technique [17] using digital buccolingual radiographs. There were no signs of calcification in the root canal, root caries, nor internal or external root resorption. The experimental conditions in our study did not require isolation. Thus, we excluded a control group in this situation. The teeth were cleaned using an ultrasonic scaler (P5 Newtron, Satelec, Acteon, France) and disinfected in 0.1% thymol (Merck, Germany), after which they were preserved in 0.5% Chloramine solution at 4 °C until future use. To prepare a 0.5% Chloramine solution, due to the presence of crystallized water in its salt structure, the amount of 0.63 g of solid Chloramine was dissolved in distilled water, bringing its volume to 10^–4^ m_3_. To prepare a solution of 0.01% of Thymol, 1 mg of solid Thymol was dissolved in 96% ethanol, bringing its volume to 10^–4^ m_3._

### Sample preparation

The teeth specimens were decoronated at the level of cemento-enamel junction under a water-cooling system to a standardized root length of 18 mm under 10 × magnification in a surgical operating microscope (Carl Zeiss OPMI Pro Ergo, Germany).

A high-speed diamond bur (Dentsply Sirona, Ballaigues, Switzerland) was used to prepare the access cavity. K-file size 10 (Dentsply Maillefer) was used in the mesiobuccal and mesiolingual canals to check patency under (10x) magnification of the dental operating microscope (Carl Zeiss OPMI Pro Ergo, Germany). The working length was measured up to 1 mm coronal to apical foramen.

Root apices of each mesial root were sealed with utility wax (Kerr, Orange, Calif.) to preserve the apical foramen from rubber base material penetration. Teeth specimens were mounted in a labeled custom-made resin 6X6 using a rubber base impression material (Kerr, Orange, Calif.), mixed according to the manufacturer's instructions, to hold and accurately position and standardize each specimen for Micro-CT. A landmark placed on the sample holder was used to guarantee the same position for pre-operative and post-operative scan. All experimental samples were scanned before instrumentation using the micro-computed tomography system (NRecon Skyscan model 1172) (Kontich, Belgium) using acquisition parameters 90 kV/112 mA. The voxel size of the data following reconstruction was 13.73 μm.

### Root canal preparation

The samples were randomly distributed according to the system (group I: 2Shape and group II: NeoNiTi) into two experimental groups. The mesiobuccal canal was used for the 2Shape system and mesiolingual canal was used for the NeoNiTi system. Both groups were subdivided according to the angle of root canal curvature into two subgroups based on whether the root canal curvature was 25° or 35°.

The preparation was conducted by one operator according to the manufacturer’s recommendation. In all groups, the instruments were discarded after single use. Endodontic motor (X-smart, Dentsply Maillefer) was used according to the manufacturer's instructions during root canal preparation for each file system. The preparation was carried out in a crown-down sequence, where the file moved with light apical pressure of about 3 mm in a slow in-and-out pecking motion and extreme torque set at 1.5 N/cm and clockwise continuous rotary motion set at 300 rpm. The process was performed again in all systems until the full working length was reached. During procedure, the canals were irrigated with 2 mL 5.25% NaOCl for 1 min between each file using a side-vented, 30-gauge needle (Ultradent, South Jordan, UT). A #10 K-file was utilized to provide apical patency between each rotary file. Paper point was used to dry the canals, after which an additional rinse with 2 mL 17% EDTA was performed after instrumentation, followed by the final irrigation using distilled water (DW; 5 mL). After irrigation, the canals were dried using absorbent paper points (Dentsply Sirona Endodontics).

### Process of micro-CT and assessment protocol

The Micro-CT device model used to obtain X-ray images of the specimens was NRecon Skyscan model 1172 Version 1.6.4 (Kontich, Belgium). For each sample, three cross-sectional slices from the apical foramen distance were chosen (before and after instrumentation) as follows: apical third considered as 3 mm distance from the anatomic apex, middle third considered as 6 mm distance from the anatomic apex, and coronal third considered as 9 mm distance from the anatomic apex [18, 19]. The volume of dentin removed for each root canal was measured in mm^3^ by subtracting the canal volume before preparation from the canal volume after preparation [20]. Canal transportation and centering ratio were calculated at 3-, 6-, and 9-mm levels from the root apex according to the formula: (× 1 − × 2)/(y1 − y2) or (y1 − y2)/(× 1 −  × 2) as described by Gambill et al. [21].

Initially, all teeth were scanned in a particular sample holder to ensure repositioning of the specimens and thus guarantee repeated measurements. Source of the X-ray was set to 90 kV/112 mA and a 0.05 mm aluminum filter was utilized to scan all specimens, to minimize scattering and artifacts of beam hardening. Each sample in the machine was rotated 360 degrees with an integration time of 5900 ms per projection. The voxel size of the data reconstructed was 13.73 μm. These settings were applied to measurements of the teeth and follow-up procedure.

CT-Analyser (CTAn) V.1.11 (Skyscan) software was applied to establish 3-D visualization and analysis of morphological parameters of the mesial canals (volume of dentin removed, canal transportation, and centering ability).

Two Micro-CT images were produced as pre-instrumentation scan, which was carried out for all evaluated teeth samples, and post-instrumentation scan. The latter scan was performed using previous parameters and positions to achieve a comparison between samples.

### Qualitative assessment

This assessment was performed by superimposing 3D images and fabricating a red-color image for an unprepared area and blue-color image for the prepared area. Furthermore, we presented cross-section of images for certain slices and clarified for each part of the experiment.

### Quantitative assessment

#### Volume of dentin removed

The canals’ pre- and post-preparation volumes were calculated separately using CT-Analyser (CTAn) V.1.11 software to fabricate a relevant 3D area of interest. The changes in canal volume for each root canal were calculated at mm^3^ by subtracting the unprepared canal volume from the prepared canal volume and comparing between the two groups.

#### Canal transportation

Direction and range of canal transportation in mm^3^ were calculated from axial sections congruous to distances of 3, 6, and 9 mm from the root apex, according to the formula presented by Gambill et al. [21]. The measurement of canal transportation was determined by comparing the distance between the unprepared canal edge to the tooth edge and the prepared canal edge to the tooth edge in mesial and distal directions.^21^ The canal transportation was calculated using the following formula: Mesiodistally = (× 1 − × 2) − (y1 − y2) as the following: **X1 and X2,** indicate the shortest distance between the mesial edge of the root to the unprepared, prepared canal edge, respectively. Meanwhile, **Y1 and Y2** indicate the shortest distance between the distal edge of the root to the unprepared and prepared canal edges, respectively.

The formula demonstrated a score of 0, signifying no canal transportation. A positive score shows mesial (lingual transportation) and a negative score shows distal (buccal transportation).

#### Centering ratio

The mean value of centering ratio is a measurement of the capability of the file to remain centered in the canal. The ratio for each section in mesiodistally direction was measured using the following formula described by Gambill et al.: (× 1 − × 2)/(y1 − y2) or (y1 − y2)/(× 1 −  × 2) [21].

The pre-and post-instrumentation scans of samples were superimposed using the software CT-Analyser (CTAn) V.1.11 in the multiplanar viewer. The formula demonstrated a score equal to 1.0, indicating preferable centralization. A ratio value closer to 0 signified that the instrument was less capable of remaining centered in the canal’s axis.

One evaluator conducted image analysis for all phases. The resultant data were stored in a database using CT-Analyser (CTAn) V.1.11 (Skyscan).

### Statistical analysis data

The mean (M) ± standard deviation (SD) values of volume of dentin removed, canal transportation, and the centering ratio were carried out using IMB SPSS software (version 23, SPSS, Inc., Chicago, Illinois, USA). For non-parametric data, the Mann–Whitney U test was utilized to determine any significant differences between the two systems for the two different morphological patterns. The level of statistical significance was set at *p* < 0.05.

## Results

### Shaping ability of root canal systems

This result discussed the effectiveness of two continuous rotation NiTi instrument systems in the shaping ability of root canal system in different canal’s morphology. The comparison between 2Shape and NeoNiTi systems manifested no significant differences regarding volume of dentin removed, canal transportation, or centering ability.

### Qualitative assessment

**Fig. 1:**
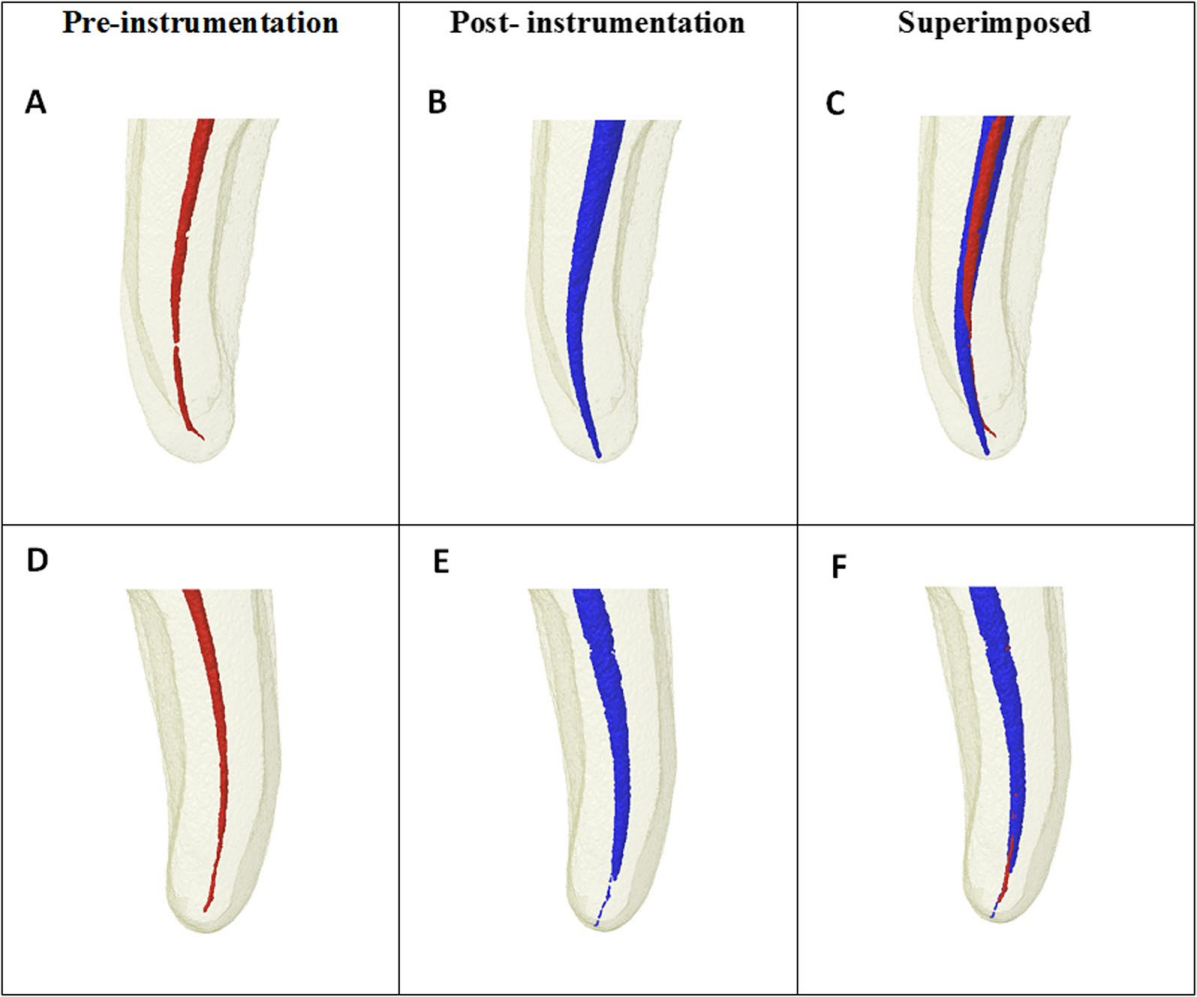
Representative 3D reconstructed image of mesiobuccal root canal prepared by 2Shape; **A** pre-preparation; **B** post-preparation and **C** superimposed, and mesiolingual root canal prepared by NeoNiTi; **D** pre-preparation; **E** post-preparation and **F** superimposed. Note canal before preparation (red) and canal after preparation (blue)

**Fig. 2:**
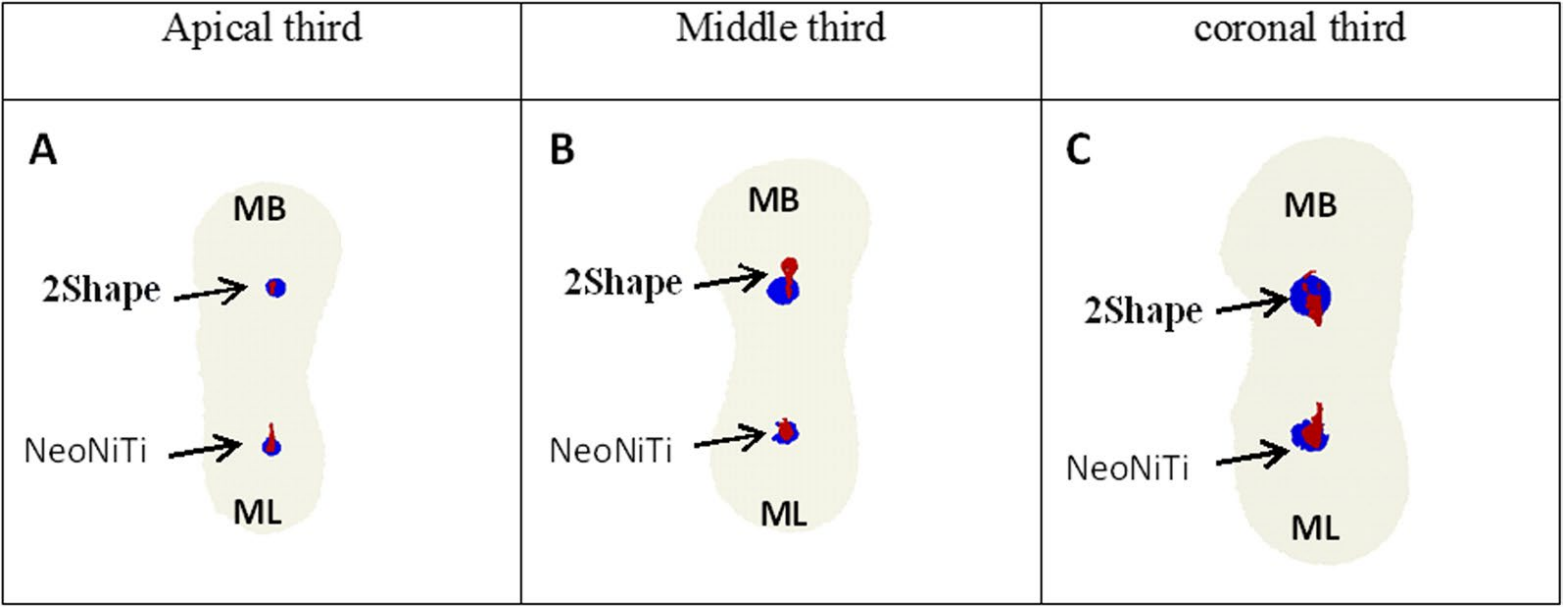
representative three-dimensional reconstructed images of root canal system for mesiobuccal and mesiolingual canals at different levels prepared by 2Shape and NeoNiTi, respectively. **A** Apical; **B** middle; and **C** coronal thirds. Note root canal before (red) and after (blue)

**Fig. 3:**
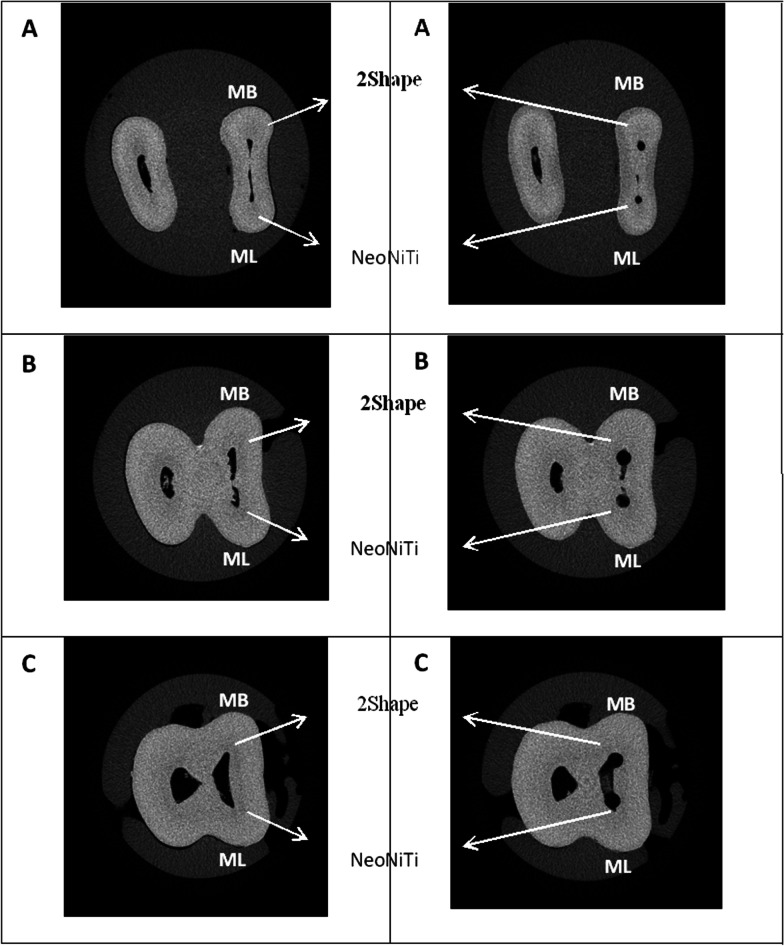
representative cross section images at different level slices of root canal system for mesiobuccal and mesiolingual canals prepared by 2Shape and NeoNiTi, respectively. Before instrumentation (left side) and post instrumentation (right side). **A** Apical; **B** middle; and **C** coronal thirds

Figure 1 provides representative Micro-CT images (A–C) of MB canal prepared by 2Shape and (D–F) of ML canal prepared by NeoNiTi, before and after preparation, and superimposed pre- and post-preparation image. Figure 2 shows illustrative Micro-CT images (A-C) at different levels (apical, middle, coronal) describing the three-dimensional reconstruction of MB canal prepared by 2Shape and ML prepared by NeoNiTi from occlusal plane. Figure 3 presents cross-section images acquired from Micro-CT image pre- and post-preparation from different level slices (apical, middle, coronal).

**Fig. 4:**
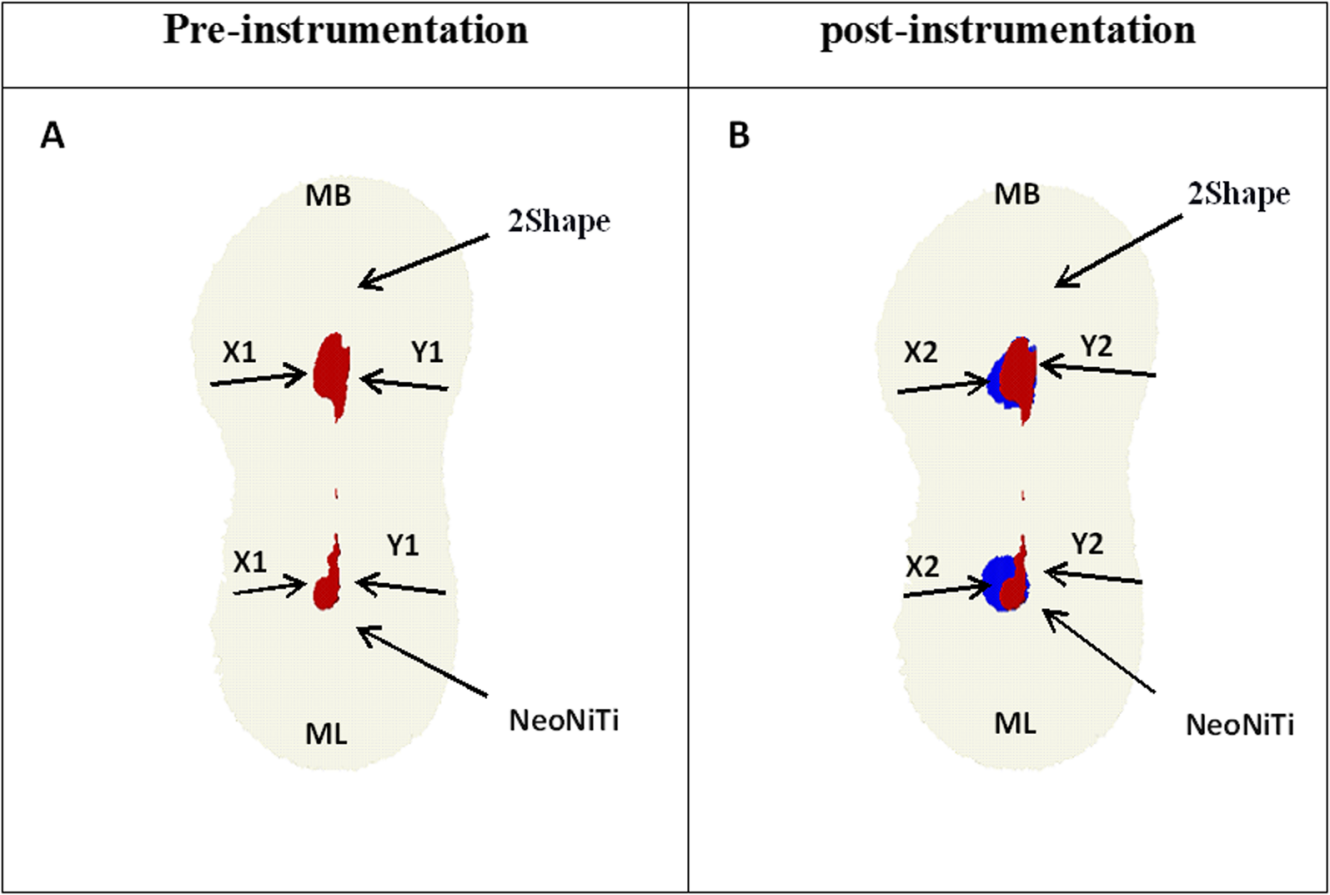
Representative 3D reconstructed images of root canal system from occlusal plane for mesiobuccal and mesiolingual canals prepared by 2Shape and NeoNiTi, respectively. **A** Canal before preparation; and **B** superimposed. X1 and X2 the distal surface of the root of unprepared and prepared canals, respectively. Y1 and Y2 the mesial surface of the root of the unprepared and prepared canals, respectively

Figure 4 represents the distance of the curved root surface from the outside to the edges of the unprepared and prepared canals (X1 and X2), respectively. Furthermore, it also represents the distance of the curved root surface from the inside to the edges of the unprepared and prepared canals (Y1 and Y2), respectively.

### Quantitative assessment

During root canal preparation, there was one file fracture from 35° root canal curvature of the 2Shape subgroup at apical third. This tooth was substituted with another one to maintain sample volume.

### Volume of dentin removed

#### Total volume of dentin removed

The (M) and (SD) values between two groups (2Shape and NeoNiTi**)** are shown in Table 1. There was no statistically significant difference in the total volume of dentin removed among the root canal for both groups (*p* = 0.557). There was no statistically significant difference in the volume of dentin removed between the two groups at different thirds (coronal third *p* = 0.557, middle third 0.398, and apical third 0.656, respectively).

**Table 1:** The Mean & standard deviation of volume of dentin removed canal transportation and centering ratio of 25° and 35° root canal curvature prepared by (2Shape & NeoNiTi)

Section	Parameter	25° 2Shape	25° NeoNiTi	*p* value^1^	35° 2Shape	35° NeoNiTi	*p* value^2^	*p* value^3^	*p* value^4^	*p* value^5^
Coronal	Volume	0.63 ± 0.37	0.68 ± 0.32	0.773	0.81 ± 0.47	0.95 ± 0.52	0.650	0.510	0.262	0.557
	Transportation	0.33 ± 0.22	0.27 ± 0.24	0.507	0.31 ± 0.19	0.32 ± 0.12	0.734	0.843	0.222	0.647
	Centering ratio	2.88 ± 1.93	2.82 ± 2.40	0.741	3.41 ± 1.66	3.65 ± 2.48	0.847	0.630	0.457	0.425
Middle	Volume	0.31 ± 0.17	0.30 ± 0.15	0.729	0.39 ± 0.28	0.54 ± 0.27	0.096	0.644	0.030*	0.398
	Transportation	0.14 ± 0.17	0.06 ± 0.10	0.106	0.09 ± 0.13	0.07 ± 0.10	0.578	0.206	0.604	0.087
	Centering ratio	3.04 ± 0.13	1.54 ± 2.02	0.078	2.42 ± 2.30	1.58 ± 1.66	0.683	0.171	0.940	0.127
Apical	Volume	0.08 ± 0.05	0.07 ± 0.03	0.525	0.09 ± 0.13	0.08 ± 0.09	0.545	0.323	0.323	0.656
	Transportation	0.07 ± 0.08	0.09 ± 0.15	0.624	0.04 ± 0.24	0.15 ± 0.19	0.364	0.792	0.644	0.725
	Centering ratio	0.80 ± 1.03	0.74 ± 0.54	0.419	1.31 ± 2.06	1.49 ± 1.82	0.762	0.553	0.843	0.411
Total	Volume	0.34 ± 0.18	0.35 ± 0.27	0.899	0.43 ± 0.27	0.52 ± 0.28	0.450	0.510	0.147	0.557
	Transportation	0.18 ± 0.08	0.14 ± 0.07	0.126	0.12 ± 0.14	0.08 ± 0.08	0.882	0.582	0.356	0.227
	Centering ratio	2.51 ± 0.96	1.87 ± 1.44	0.149	2.38 ± 1.08	2.02 ± 1.06	0.916	0.234	0.501	0.280

^1^*p* value using Mann–Whitney U between 25° 2Shape and 25° NeoNiTi

^2^*p* value using Mann–Whitney U between 35° 2Shape and 35° NeoNiTi

^3^*p* value using Mann–Whitney U between 25° 2Shape and 35° 2Shape

^4^*p* value using Mann–Whitney U between 25° and 35° NeoNiTi

^4^*p* value using Mann–Whitney U between NeoNiTi and NeoNiTi

*p value < 0.05

#### 25° root canal curvature at the different root thirds

Regarding total volume of dentin removed, there was no statistically significant difference in the volume of dentin removed among the whole root canal for both groups of 25° root canal curvature (*p* = 0.899) (Table 1). Additionally, there was no statistically significant difference in the volume of dentin removed between the two groups at the different thirds (coronal third *p* = 0.773, middle third 0.729, and apical third 0.525, respectively).

#### 35° root canal curvature at the different root thirds

There was no statistically significant difference in the volume of dentin removed among the whole root canal for both groups of 35° root canal curvature (*p* = 0.450) (Table 1). There was also no statistically significant difference in the volume of dentin removed between the two groups at different thirds (coronal third p = 0.650, middle third 0.096, and apical third 0.545, respectively).

#### 2Shape 25° and 35° root canal curvature at different thirds

There was no statistically significant difference in the volume of dentin removed between 25° and 35° root canal curvature at different thirds within the subgroups (coronal third *p* = 0.510, middle third 0.644, and apical third 0.323, respectively) (Table 1).

#### NeoNiTi 25° and 35° root canal curvature at different thirds

There was a statistically significant difference in the value of dentin volume removed between 25° and 35° root canal curvature at the middle third (*p* = 0.030) (Table 1). On the other hand, at the coronal and apical thirds, there was no statistically significant difference in the volume of dentin removed between 25° and 35° root canal curvature within the subgroups (coronal third p = 0.262 and apical third 0.323, respectively).

### Canal transportation

There was no statistically significant difference in the amounts of mesiodistal transportation between all the parameters investigated for volumes at different thirds (Table 1).

### Centering ratio

There was no statistically significant difference in the total centering ratio of mesiodistal direction among the root canal between all the parameters investigated for volumes at different thirds (Table 1).

## Discussion

The potential of canal transportation during root canal preparation can be reduced by utilizing advanced metallurgy of rotary systems to improve the canal preparation, preserve the canal geometry, and minimize the incidence of mishaps [22]. Several issues may arise during canal preparation procedures, such as perforation, ledge formation, canal blockage, elbow, canal transportation, and a broken file. The systems used in this study were designed to reduce the procedural errors and enhance canal preparation shape. Micro-CT was chosen to assess the alterations in the canal shaping and facilitate comparison between pre- and post-preparation [21, 23, 24]. CT scans are also used in the endodontics field to assess the preparation of root canals using stainless steel hand endodontic instruments and nickel-titanium (NiTi) and compare the endodontic features of dentin removal, canal transportation, and centralization of the files after canal preparation [21].

In the present study, we highlighted the effectiveness of a newly designed instrument representing a new generation of rotary systems in shaping changes in root canal. We focused on two relatively new rotary nickel titanium with similar kinematics during instrumentation and a different cross-section, manufactured by proprietary heat treatment and used as single-file nickel titanium system that required only one instrument for shaping the root canal. Therefore, preparation requires less time than full-sequence rotary instruments [25].

The 2Shape instrument (TS) system is made from (T-wire) technology, which enhances the flexibility of instruments and fractures resistance [10, 11]. The instrument consists of two main and one secondary cutting edge and non-cutting safety tip for better negotiation of canal curvature. The two cutting edges demonstrated an excellent cutting performance, and the secondary cutting edge enhanced the removal of debris.

On the other hand, NeoNiTi instruments are a newly introduced nickel titanium rotary system made from a controlled memory (CM) nickel titanium wire and manufactured by electrical discharge machining (EDM) [26]. This method contains some considerable advantages. For instance, the rough surface can enhance the cutting efficiency of the file and heat treatment, resulting in high flexibility and increased cyclic fatigue resistance [27].

Shaping ability was evaluated based on three parameters that significantly affect the success of root canal therapy: the volume of dentin removed, canal transportation, and centering ability. The volume of dentin removed from the canal’s 3D geometry were evaluated separately at three different levels to determine the differences in canal shape after preparation of the interested area.

Canal transportation match to the post instrumentation deviation in the axis was similar to the original pre-instrumentation axis. The American Association of Endodontists clarified transportation of the canal as “excessive removal of the outside curve of the canal wall that occur because of the orientation of the files to return to their primary shape” [28]. Centering ability refers to the capability of the file axis to align with the canal center, thereby avoiding canal ledging, zipping, or perforation during root canal treatment [29].

In maintaining the torsional properties of M-Wire, the alloy has to be significantly more cyclically fatigue-resistant than conventional NiTi alloy [30]. The improved fatigue resistance is attributed to a developed resistance of fracture beginning related to the reorientation ability of the martensitic phase variations [31].

KT Ceyhanli et al*.* used stereoscopic images to compare the effects of shaping between two types of NiTi alloy, traditional (ProTaper Universal, Sendoline S5), and M-Wire (WaveOne, GT series X). The manufacturing processes (M-Wire or traditional NiTi) were not found to influence the shaping capability of the systems. The small tapered file systems design resulted in lower deviations of the file during root canal preparation and less removed of material than highly tapered NiTi systems [32].

Zhi Cui et al*.* used M-CT to assess the shaping ability of two NiTi instrument systems (ProTaper Next and Wave One) in S-shaped canals. They found that there was no significant difference between the two systems at the cervical third. However, the WaveOne system at the apical third caused more transportation than the ProTaper Next system [33].

CM Wire files have a significantly improved resistance to cyclic failure than conventional and M-Wire NiTi instruments [34], which might be attributed to the failure of a deflection angle is lower than conventional and M-Wire NiTi [35].

Mozzami et al. assessed the canal transportation of two systems of NiTi instruments (CM Wire: NeoNiTi and M Wire: Reciproc) using (CBCT) as an evaluation method. They realized a significant difference between the systems regarding canal transportation. NeoNiTi produced less transportation than Reciproc in both mesiodistal and buccolingual orientations [36].

Forghni et al. assessed the root canal shaping ability of NeoNiTi and Protaper Universal nickel-titanium endodontic instrument systems using superimposed digital images ofseverely curved simulated canals.The results showed that no significant differences among the systems in changes of canal angle. However, the canal deviation of the Neolix system showed less than the protaper Universal system [37].

In our study, extracted first molars of mandibular teeth with severe mesial root curvatures were used to simulate clinical conditions, rather than simulated artificial canals, due to the natural anatomical features and dentin properties of root canal teeth.

Two separate canals were selected because they were narrow and curved, which increased the difficulty of instrumentation [38]. The samples had similar preoperative geometric parameters, namely, initial apical diameter (K file #10), length characteristic (18 mm), and angle of curvature (25° and 35°). Measurement of the root curvature of the teeth was achieved using Schneider’s technique [17], which is the most common method of measuring root canal curvature in the literature [39]. The curvatures of all root canal were 25° and 35° to adequately standardize the experimental groups, as curvature can influence the shape of canal preparation [40]. The teeth were then randomly distributed between groups (2Shape and NeoNiTi) based on root canal curvature. We used both systems in the same specimen (mesiobuccal and mesiolingual) to standardize the comparison between the two systems.

No articles were found in the literature that used the same two standardized angles as our study. Instead, all previous studies (e.g., Capar et al.) showed angles at a severe curvature, between 20° and 40°.

Sodium hypochlorite (NaOC1) (5.25%) was used as the irrigation solution during chemo-mechanical preparation, at the most common concentration used in routine endodontic therapy. A 30-gauge needle size tip was inserted up to 1–2 mm short of the length to facilitate the irrigant’s introduction to the apical third [41], followed by 2 ml of 17% EDTA after instrumentation to simulate the clinical conditions [41, 42]. To obtain comparable results, the apical preparation diameter was similar in all samples to size 25 represented by TS2 in the 2Shape group and A1 in the NeoNiTi group [43].

In this study, the prepared canal was evaluated at three levels: 3, 6, and 9 mm from the root apex (apical, middle, and coronal thirds) [44]. These levels are highly susceptible to iatrogenic mishaps [40]. Micro-computed tomography (Micro-CT) was chosen because it is considered as the ideal method for laboratory studies: its practicality, accuracy, reliability, reproducibility, and non-destructiveness allow for effective qualitative and quantitative 3D evaluation of root canals [45].

In the present study, we observed a significant difference in volume of dentin removed between 25° and 35° canal curvatures in the NeoNiTi system. The NeoNiTi reported relatively more volume of dentin removed in the middle third at 35° canal curvature than at 25°. This could be attributed to a homothetic rectangle cross-section; taper size of A1 NeoNiTi file (0.8); circumferential brushing motion in the coronal and middle third; hard cutting edges; and an abrasive property of the flutes [12], that may cause excessive cutting, thereby aggressing the dentin volume [46]. Meanwhile, 2Shape file at 35° curvature compared to 25° did not reveal the same result in dentin volume cutting. Subsequently, we noted that the 2Shape group did not exceed that removal of dentin volume. These results are in consistent with previous articles [47, 48].

Singh et al*.* evaluated the volume of dentin removed, canal transportation, and centering ability of 2Shape (2S) and WaveOne Gold (WOG) at different root levels using cone-beam computed tomography (CBCT). They conclude no significant difference between the NiTi files at various root canal levels [49]. Our study assessed and compared the volume of dentin removed, canal transportation, and centering ability at 3, 6, 9 from the apical foramen, and found that neither 2Shape nor NeoNiTi caused significant transportation.

Similarly, Singh et al. evaluated the volume of dentin removed with 2Shape and Protaper Gold in severe root curvature using CBCT. Results found a significant difference between the 2Shape and ProTaper Gold systems at the 3 mm, 5 mm, and 7 mm level. They concluded that the 2Shape file has the less aggressive removal of dentin [48]. This finding supports our experimental results reporting less volume of dentin removed.

An ex vivo study of the shaping ability evaluation of 2Shape, NeoNiTi, and Protaper Next systems in severely curved root canals at different canal levels was conducted by Hussien et al*.* They concluded no statistically significant difference in amounts of mesiodistal canal transportation between the systems at three canal levels. All the files system showed some degree of canal transportation. [50].

The results of the present study demonstrated that both rotary systems had comparable values of canal transportation and centering ability among the canal levels. The NeoNiTi files showed less canal aberrations compared to 2Shape files. These findings might be attributed to the improvement in flexibility of the NeoNiTi instruments due to CM Wire technology and manufactured (EDM) method, which makes the file respect the canals anatomy and improves negotiation of curvatures [51, 52].

In the 2Shape group, one case of broken instrument occurred which did not occur in the NeoNiTi group. Moreover, the 2Shape system design features a triple-helix cross-sectional, whereas the NeoNiTi system has a homothetic-rectangle cross-section. The design of the NeoNiTi file is likely associated with the increased fatigue resistance compared to the 2Shape file. Consequently, using this system seems to be safer regarding file breakage. These findings are in conformity with the results of Ozyurek et al. [53], who found that the 2Shape within canal curvatures showed a significantly lower resistance to cyclic fatigue.

Wu et al. reported transportation at apical third greater than 0.3 mm, thus indicating a capability of adversely influencing the sealing ability of root canal filling materials and treatment prognosis [54]. In the present study, the amount of canal transportation for the two evaluated systems (2Shape and NeoNiTi) remained beneath this limit.

The key message for the clinicians is that when used clinically, the NeoNiTi system may perform better and produce more centered preparation and minimal canal deviation compared to the 2Shape system. Consequently, the null hypothesis in our study was rejected. However, the present study contains several limitations. For instance, it was performed on extracted teeth and thus did not fully represent the conditions present in the oral cavity that might produce different angulation and inclination, which would in turn affect the root canal preparation. Further in-vivo studies are necessary to accurately assess the effect of file designs on the shape of root canals with different morphological patterns.

## Conclusions

Within the limitations of our in vitro study, we conclude that both 2Shape and NeoNiTi systems with severely curved canals were relatively safe in preparation and respected original canal anatomy. Nevertheless, NeoNiTi instruments produced more centered preparation and minimal canal deviation compared to the 2Shape system.

## Data Availability

All co-authors agree to disclose publicly for all available datasets presented in the main paper.

## Abbreviations

NiTi: Nickel-titanium
Micro CT: Micro Computed Tomography
EDM: Electrical discharge machining
CM: Controlled Memory
NaOCl: Sodium Hypochlorite
